# Porcelain Gallbladder

**DOI:** 10.5005/jp-journals-10018-1244

**Published:** 2017-09-29

**Authors:** Apoorv Goel, Ayush Agarwal, Shalabh Gupta, Tripta S Bhagat, Gaurav Kumar, Atul K Gupta

**Affiliations:** 1 Department of General Surgery, Santosh Medical College and Hospital, Ghaziabad, Uttar Pradesh, India

**Keywords:** Carcinoma gallbladder, Chronic cholecystitis, Porcelain gallbladder, Prophylactic chole-cystectomy.

## Abstract

Porcelain gallbladder or calcified gallbladder is a rare entity and is considered as the end stage of chronic cholecystitis. This disease is rarely diagnosed preoperatively and usually mimics carcinoma gallbladder. Hereby, we present a rare and interesting case of porcelain gallbladder that was diagnosed preoperatively and managed by cholecystectomy.

**How to cite this article:** Goel A, Agarwal A, Gupta S, Bhagat TS, Kumar G, Gupta AK. Porcelain Gallbladder. Euroasian J Hepato-Gastroenterol 2017;7(2):181-182.

## INTRODUCTION

Porcelain gallbladder is an extremely rare condition with an incidence of 0.06 to 0.8% and considered as high risk for carcinoma gallbladder.^1,2^ The risk for malignancy ranges from 5 to 22%.^3^ It is also known as calcified gallbladder and is considered as end stage of chronic cholecystitis. It is more common among elderly females and due to high risk of malignant transformation it is managed by cholecystectomy.

## CASE SUMMARY

**Fig. 1:**
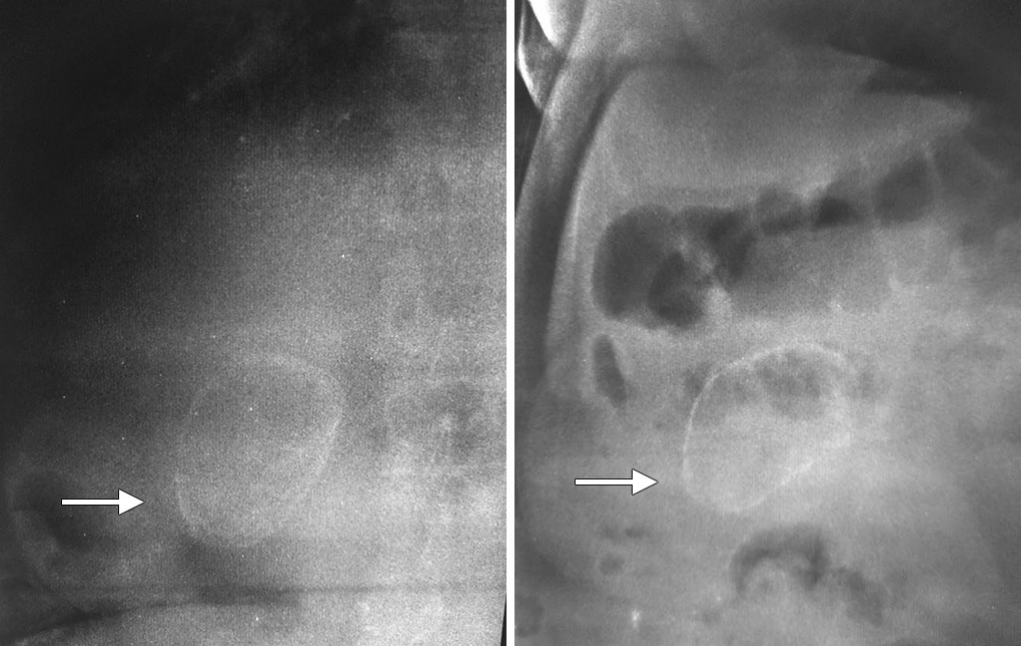
Plain X-ray abdomen showing calcified gall bladder

**Fig. 2:**
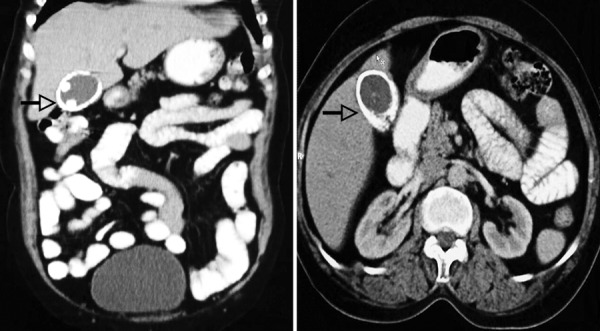
Contrast-enhanced computed tomography of abdomen

A 55-year-old female presented with complaints of intermittent pain in right upper abdomen of 15 days duration. There was no history of any fever, jaundice, and vomiting. Abdominal examination was unremarkable. Ultrasound abdomen revealed a large calcified mass in gallbladder fossa with normal common bile duct and normal liver echotexture. After corroboration with X-ray abdomen (Fig. 1), the provisional diagnosis of porcelain gallbladder was made. Contrast-enhanced computed tomography (CECT) abdomen confirmed the diagnosis of porcelain gallbladder with no evidence of malignant change (Fig. 2). Liver function test was normal. Patient was planned for open cholecystectomy. Intraopera-tively, it was a difficult dissection as gallbladder was completely calcified, brittle, and densely adhered to liver bed. After dissecting Calot’s triangle and dividing cystic duct and artery, gallbladder was excised and sent for histopathologic examination. Postoperative recovery was uneventful.

Histopathology report revealed rigid, thickenec chronically inflamed gallbladder wall with transmura calcification. Gallbladder was packed with multipl stones. Microscopic examination showed hemorrhagi and fibrosis with focal muscular hypertrophy and cal cification. There was no evidence of malignant change.

## DISCUSSION

Porcelain gallbladder or calcified gallbladder or cholecys topathia chronica calcarea is a relatively rare condition with an incidence rate of 0.06 to 0.8%.^1,2^ It is characterized by bluish discoloration and extremely calcified wall of gallbladder. It is commonly seen among elderly females and is associated with gallstones in about 90% of cases.^1-3^ It is associated with high risk of malignancy and the rate may vary from 5 to 22%.^3,4^ Porcelain gallbladders are associated with gallstones in 90% of cases. The patho-genesis of gallbladder calcification is still unclear and it is considered a result of obstruction of cystic duct leading to the precipitation of calcium salts in the mucosa or as a result of chronic inflammation resulting in the hemorrhage, scarring, and hyalinization of the wall causing deposition of lime salts. Histological examination shows that the calcification is diffusely scattered throughout the mucosa, submucosa, glandular spaces, and Rokitansky-Aschoff sinuses.^1,2,4^ This chronic inflammation or the degeneration and regeneration process within the gallbladder epithelium may act as a carcinogenic stimulus.

Porcelain gallbladder patients are usually asymptomatic and this condition is diagnosed as an incidental finding on plain abdominal radiograph, sonography, and CT.^5^ There are several conditions like large opaque gallstone, calcified hydatid cyst of liver, metastatic deposits, calcified renal cysts, schistosomiasis, calcified lymph nodes, and other granulomatous diseases, which may closely mimic porcelain gallbladder. However, CT is considered as investigation of choice as it can accurately differentiate it from other causes.^5^

Porcelain gallbladder is associated with a high risk of malignancy; hence, surgical removal should not be delayed. Prophylactic cholecystectomy should be performed.^6-8^ Open cholecystectomy is preferred over laparoscopic approach due to suspicion of malignancy as well as brittle consistency of gallbladder; however, few studies have quoted successful laparoscopic chole-cystectomy in porcelain gallbladder.^6-8^

## CONCLUSION

Porcelain gallbladder is a rare condition and associated with high risk of carcinoma gallbladder. Prophylactic cholecystectomy is the preferred treatment for porcelain gallbladder.
